# Jumonji C Demethylases in Cellular Senescence

**DOI:** 10.3390/genes10010033

**Published:** 2019-01-09

**Authors:** Kelly E. Leon, Katherine M. Aird

**Affiliations:** Department of Cellular & Molecular Physiology, Penn Stage College of Medicine, Hershey, PA 17033, USA; kleon2@pennstatehealth.psu.edu

**Keywords:** epigenetics, cell cycle, metabolism, histones, senescence-associated secretory phenotype, senescence-associated heterochromatin foci, p53, retinoblastoma protein

## Abstract

Senescence is a stable cell cycle arrest that is either tumor suppressive or tumor promoting depending on context. Epigenetic changes such as histone methylation are known to affect both the induction and suppression of senescence by altering expression of genes that regulate the cell cycle and the senescence-associated secretory phenotype. A conserved group of proteins containing a Jumonji C (JmjC) domain alter chromatin state, and therefore gene expression, by demethylating histones. Here, we will discuss what is currently known about JmjC demethylases in the induction of senescence, and how these enzymes suppress senescence to contribute to tumorigenesis.

## 1. Introduction

Chromatin is organized through nucleosomes, which contain an octamer core of wrapped DNA [1,2]. This octamer core consists of two copies of each core histone, H2A, H2B, H3, and H4 [2,3]. Core histones are primarily identified by their histone fold domain and their N-terminal tails [4]. Interestingly, histone tails undergo various post-translational modifications such as acetylation, methylation, phosphorylation, ubiquitination, and others [5,6]. These various modifications can result in gene expression alterations through changes in electrostatic charge, which alters DNA accessibility [7].

A common histone modification that can result in changes to gene expression is methylation, a reversible post-translational modification [8,9]. Histone methylation occurs on lysine, arginine, and histidine residues, resulting in transcriptional activation or silencing of genes, the recruitment of DNA damage response proteins, and changes in chromosomal packaging and DNA accessibility [10,11]. Through the recruitment of various enzymes, such as histone methyltransferases (HMTs) and histone demethylases, the methylation state of a particular histone can be altered. HMTs have been extensively reviewed elsewhere [12]. Here, we will focus on a conserved group of histone demethylases containing a Jumonji C (JmjC) domain [8,13,14]. JmjC demethylases are oxygenases dependent on Fe(II) and α-ketoglutarate (αKG) for their activity [8,13,15,16]. Histone demethylation through JmjC proteins occurs through a hydroxylation reaction, in which αKG, oxygen, and Fe(II) are used to produce succinate and CO_2_ [12,13,15]. This hydroxylation reaction results in unstable hemiaminal products, which are broken into formaldehyde and a demethylated histone product [13,15]. Twenty-four human enzymes are currently known to contain a JmjC domain and have histone demethylase activity [17]. These enzymes are organized into subfamilies based on sequence homology and methylation state preference [17,18].

JmjC histone demethylases regulate chromatin state through the removal of mono-, di-, and tri- methylation marks on the lysine residues of multiple histones [19]. Changes within the chromatin state can lead to either activation or repression of transcription, depending on the amino acid residue that is targeted. Additionally, methylation changes may also affect other cellular processes, such as DNA damage repair, without affecting transcription of surrounding genes [20,21]. Consistently, JmjC demethylases play a role in various pathological conditions [17,22]. For instance, JmjC demethylases have been shown to be both pro-tumorigenic and tumor suppressive [23,24]. This is due in part to their activity in inducing and suppressing senescence, a stable cell cycle arrest that is primarily mediated by the tumor suppressors p53 and pRb (retinoblastoma protein) [25,26]. Here we will discuss the role of JmjC demethylases in senescence in the context of suppressing or promoting tumorigenesis.

## 2. Role of Jumonji C Demethylases in Senescence

A number of studies have shown a role for JmjC demethylases in senescence. Here, we will discuss what is known about these enzymes (specifically KDM6B, KDM5A, KDM5B, KDM4A, KDM4B, and KDM2B) in regulating the senescence phenotype (Table 1).

### 2.1. Cellular Senescence

Cellular senescence is defined as a stable cell cycle arrest [25]. Senescence can occur due to a variety of stimuli, including oncogenic stress [26,43]. Therefore, senescence is considered a tumor suppression mechanism [44]. Two canonical tumor suppressor pathways play a role in maintaining cells in the senescence-associated cell cycle arrest, p53/p21 and p16/pRb [26,45]. Senescent cells have a marked change in their epigenome, in part through increased repressive histone modifications, in particular H3K9me3/2 [46]. This along with other changes in the chromatin structure of senescent cells is called senescence-associated heterochromatin foci (SAHF) [46,47]. Increased repressive H3K9me3/2 is observed at proliferation-promoting E2F gene targets such as *CCNA2*, *PCNA*, *MCM3*, and *DHFR* [48]. This inhibits transcription of these genes and in part promotes the senescence phenotype [46,48].

**Figure 1:**
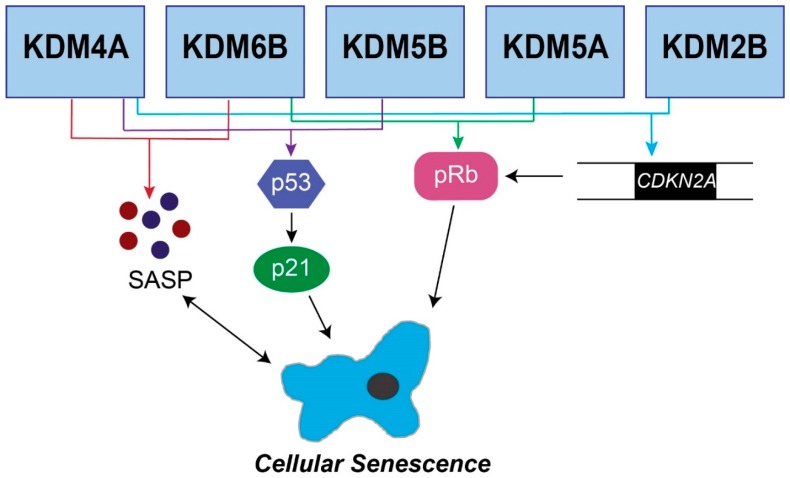
Senescence pathways regulated by JmjC demethylases. The five JmjC demethylases shown are known to affect senescence through the p16/pRb pathway, the p53/p21 pathway, or the senescence-associated secretory phenotype. KDM6B, KDM5B, KDM5A, KDM4A, and KDM2B have all been shown to regulate *CDKN2A*, which feeds into the pRb pathway. Additional JmjC demethylases (KDM6B, KDM5B, and KDM5A) directly regulate pRb. Multiple enzymes regulate the p53/p21 pathway to induce senescence, including KDM6B, KDM5B, and KDM5A. Finally, KDM4A and KDM6B regulate the SASP.

In addition to SAHF, senescent cells also acquire a unique microenvironment known as the senescence-associated secretory phenotype (SASP). SASP gene transcription is increased during senescence, resulting in an increase in cytokines, chemokines, and matrix metalloproteinases (MMPs) in the senescent microenvironment [28,49]. The increase in inflammatory cytokines due to the SASP can have detrimental side effects resulting in chronic inflammation and tumorigenesis [49,50]. In contrast, the SASP contributes to the clearance of senescent cells, thereby limiting tumorigenesis [51]. JmjC demethylases have been shown to affect both the SAHF and SASP during senescence (Figure 1), which will be addressed in detail below.

### 2.2. KDM6B

KDM6B demethylates the repressive mark lysine 27 on histone H3 (H3K27me3) [52]. KDM6B is important for multiple senescent phenotypes, including SASP gene expression, p16 expression, SAHF, and p53 expression [27,28,29,53]. Overexpression of KDM6B increases SASP gene expression in glioma cell lines [27]. Consistently, H3K27 methylation is lost at SASP gene loci, suggesting that H3K27 methylation suppresses SASP gene expression under normal conditions. In addition to increasing SASP gene expression, other studies have demonstrated that KDM6B expression increases during cellular senescence, and this may in part help maintain the *CDKN2A* locus (encoding p14^ARF^ and p16^INK4A^) in a demethylated and activated state [29,53]. Moreover, KMD6B demethylates retinoblastoma protein (pRb), which inhibits its interaction with cyclin-dependent kinase 4 (CDK4) and reduces pRb phosphorylation formation [28]. pRb is known to play a role in SAHF formation [46]. Therefore, KDM6B promotes SAHF formation through demethylating pRb. Finally, KDM6B regulates p53 by binding to p53-responsive promoter and enhancer elements upon DNA damage [29]. Although the mechanism is not clear, it is interesting to speculate that KDM6B is necessary at those loci to remove H3K27 methylation so that DNA damage response genes are not repressed. In this context, KDM6B expression may allow for cells to overcome DNA damage-induced senescence. Therefore, further work needs to be performed to fully understand whether KDM6B is tumor suppressive or tumor promoting. Together, these findings suggest that KDM6B is important for multiple senescence pathways. It will be interesting in the future to determine whether KDM6B activity acts in concert at multiple loci during senescence to affect this phenotype.

### 2.3. KDM5B

KDM5B, also known as JARID1B, demethylates lysine 4 on histone H3 (H3K4me), an active histone mark [54]. Two distinct methods for induction of senescence through KDM5B activity have been described. Upon knockdown of KDM5B, colorectal cancer (CRC) cells have increased H3K4 methylation at the *CDKN2A* locus [30]. This correlates with decreased proliferation and increased senescence-associated-β-galactosidase (SA-β-Gal) activity, a marker of senescence [30,55]. Additionally, KDM5B, through a direct interaction with pRb, promotes H3K4me3/2 demethylation in a model of oncogene-induced senescence, which results in the silencing of E2F target gene promoters [31]. Consistently, overexpression of KDM5B decreased H3K4 methylation and proliferation-promoting E2F gene targets and increased senescence. Together, these two studies demonstrate that H3K4 methylation is important for proliferation-promoting gene expression to suppress senescence, and modulation of H3K4 demethylase KDM5B affects both expression of these genes and the senescence-associated cell cycle arrest.

Additionally, many reports indicate that the demethylase activity of KDM5B is critical for DNA damage repair and genomic instability [56,57,58]. Indeed, inhibition or knockdown of KDM5B activates p53 and inhibits cell proliferation [56,57], hallmarks of senescence. Therefore, it is possible that cells with KDM5B knockdown are undergoing senescence due to an alteration in the DNA damage response. Future work will need to determine the role of KDM5B loss in the DNA damage accumulation observed during senescence.

### 2.4. KDM5A

Similar to KDM5B, KDM5A (JARID1A/RBP2), demethylates H3K4me3/2 [13]. KDM5A binds to pRb to modulate transcription downstream of pRb [59]. Knockdown of KDM5A induces senescence through an increase in cyclin-dependent kinase inhibitors such as p21, p27, and p16 in multiple cell types through increased H3K4me3 occupancy at the promoters of these genes [32,33]. pRb is critical for SAHF formation during senescence [46]. Thus, future studies are needed to determine the role of KDM5A–pRb binding in SAHF formation.

### 2.5. KDM4A

KDM4A demethylates H3K9me2 and H3K9me3, two repressive histone marks [60]. Additionally, the co-crystal structures of KDM4A suggest KMD4A also recognizes and binds to H3K4me3/2 and H3K20me3/2 active and repressive marks, respectively [61,62]. Downregulation of KDM4A activates the p53 pathway, thereby inducing senescence [35]. Specifically, loss of KDM4A increases repressive H3K9me3 at the promoter region of chromodomain helicase DNA binding protein 5 (*CHD5*), resulting in transcriptional repression of *CHD5* [63]. This in turn upregulates p53 levels to induce senescence. In addition, knockdown of KDM4A induces an accumulation of promyelocytic leukemia (PML) bodies [35]. PML bodies, a marker of senescence, regulate SASP gene expression and fuse with persistent DNA damage foci termed DNA segments with chromatin alterations reinforcing senescence (DNA-SCARS) [64,65]. While the authors did not investigate the role of KDM4A on SASP gene expression, it is possible that the loss of KDM4A may also affect transcription of these genes through the active H3K4 histone mark. Another study found that KDM4A forms a complex with F-box protein 22 (FBXO22) to target methylated p53 for ubiquitin-mediated degradation [36]. Consistent with the study by Mallette et al., this study showed that depletion of KDM4A upregulates p53 expression and induces senescence. Interestingly, this study also found that the FBXO22–KDM4A complex is necessary for p16 and SASP induction in late-stage senescence. However, a role for KDM4A as a histone demethylase in this context is unclear. Finally, microRNAs have been shown to regulate *KDM4A* gene expression by binding to its 3′ UTR. In particular, miR-10a has been shown to bind and repress *KDM4A*, resulting in growth arrest of prostate cancer cells [66]. Additionally, decreased KDM4A expression, mediated through miR-137, leads to an induction of senescence through the p53 and pRb pathway. However, it is unknown whether this is through histone demethylation or other mechanisms. Together, these studies demonstrate that KDM4A expression and activity are important for both p53 and p16 expression and that inhibition of KDM4A induces senescence.

### 2.6. KDM4B

Similar to KDM4A, KDM4B also demethylates H3K9me3 [67]. Although it has not been shown to directly affect the induction of senescence, KDM4B is in part regulated by p53 [37,38], a critical senescence modulator [25]. *KDM4B* expression is activated upon p53 binding to its promoter region [63]. When this occurs, p53 no longer efficiently activates the expression of its downstream targets *p21*, *PIG3*, and *PUMA*. Interestingly, KDM4B was not recruited to the promoters of *p21*, *PIG3*, or *PUMA*, and H3K9me3 remained high at these loci. Further work will be needed to elucidate how KDM4B is indirectly affecting the DNA damage response downstream of p53. Additionally, KDM4B is important for repair of DNA double strand breaks [38,39], and knockdown of KDM4B inhibits proliferation of cancer cells after induction of DNA damage [38]. Together, these data may explain why KDM4B is high in cancer cells [68], to limit the effects of p53 and promote DNA repair to overcome or bypass senescence.

### 2.7. KDM2B

KDM2B is a conserved nuclear protein that demethylates both active histone marks (H3K4me3, H3K36me2, and H3K79me3/2) and the repressive histone mark H3K27me3 [69]. Multiple studies have demonstrated that KDM2B demethylates H3K4me3 and H3K36me2 at the *CDKN2A* and *CDKN2B* loci [40,41]. Consistently, increased KDM2B immortalizes MEFs [24,40]. Recent work identified KDM2B as the histone demethylase for H3K79. A deficiency in DOT1L, the H3K79 histone methyltransferase, induces senescence [70]. Future work will need to determine whether KDM2B affects this mark during senescence. Together, these studies emphasize the critical role of KDM2B in gene silencing and preventing cellular senescence.

Overall, JmjC demethylases play a role in cellular senescence by targeting proteins involved in one of the two major tumor suppressive pathways involved in maintaining cells in a senescence-associated cell cycle arrest, namely p16 and p53. As cellular senescence is both beneficial and detrimental, further investigations into how JmjC demethylases affect senescence and especially the SASP will be essential for understanding whether these enzymes can be targeted for therapeutic purposes to inhibit or eliminate senescent cells.

## 3. Jumonji C Histone Demethylases Suppress Senescence during Tumorigenesis

### 3.1. Jumonji C Demethylases Affect Cell Cycle Regulatory Genes to Suppress Senescence

As discussed above, senescence is defined as a stable cell cycle arrest [25]. Therefore, it is thought to be a bona fide tumor suppressor mechanism, and overcoming senescence is a critical barrier in tumorigenesis in some tumors [71,72]. Numerous JmjC demethylases are associated with tumorigenesis through a role in suppressing senescence (Table 1). This is mostly due to modulation of cell cycle-related genes and the p53 pathway, as detailed below.

KDM5B and KDM5A are highly expressed in cancer [73,74]. However, only KDM5A has been shown to play a role in suppressing senescence during tumorigenesis. As mentioned above, KDM5A binds to pRb to promote senescence [59]. In Rb-null tumors, KDM5A ablation prolongs survival of mice, which correlates with increased H3K4me3 [34]. While the authors did not show that KDM5A overexpression induces tumorigenesis, it is interesting to speculate that increased KDM5A activity alone may overcome senescence.

KMD2B is overexpressed in multiple cancer types [75]. In regards to suppressing senescence, KDM2B expression plays a role in pancreatic cancer tumorigenesis by bypassing KRAS^G12D^-mediated senescent lesions (pancreatic intraepithelial neoplasia) [42]. Additionally, Kdm2b expression immortalizes MEFs by suppressing replicative senescence through inhibition of both the pRb and p53 pathways [24]. This suggests that KDM2B may play a role in tumorigenesis through inhibiting these two critical tumor suppressor pathways. Interestingly, KDM2B expression alone is not able to suppress replicative senescence in human cells, suggesting that it cannot protect cells from telomere erosion [24]. Finally, KDM2B cooperates with HRAS^G12V^ to facilitate transformation of MEFs [41].

KDM4A cooperates with RAS to promote transformation through suppression of the p53 pathway [35]. It is possible that KDM4A is necessary to suppress senescence as it is highly expressed or amplified in a number of human cancers [68,76,77]. Together, it is clear that some JmjC demethylases are critically important for suppressing senescence. Future work will need to determine the specificity of the JmjC demethylase and its downstream targets during tumorigenesis.

### 3.2. JmjC Demethylases Affect the Senescence-Associated Secretory Phenotype

In addition to the cell cycle arrest, senescence is also often defined by the SASP [78]. The SASP can be tumor suppressive or tumor promoting, depending on the context [49]. Although the SASP can result in the clearance of tumorigenic cells through an immune response [51], it can also promote tumorigenesis through its detrimental side effects [49]. For instance, the SASP can induce epithelial-mesenchymal transition (EMT), mediated primarily through the increase in IL-6 and IL-8 [78,79]. In addition, the SASP increases angiogenesis through vascular endothelial growth factor (VEGF) expression [80]. Finally, the SASP can promote chemotherapy resistance [49]. As some JmjC demethylases affect SASP gene expression, it is possible that these demethylases contribute to tumorigenesis by promoting chronic inflammation. For instance, KDM6B overexpression in glioma cells increases SASP gene expression, leading to tumorigenesis and tumor progression [27]. Additionally, KDM4A is necessary for the SASP as knockdown of KDM4A in combination with RAS expression inhibits *IL6* and *IL8* expression [36].

As more research comes to light about histone methylation in DNA damage repair and gene transcription of the SASP and cell cycle regulators, in addition to non-histone targets of JmjC demethylases, we will undoubtedly learn more about their roles in tumorigenesis.

## 4. Targeting JmjC Demethylases for Cancer Therapy

As we have discussed here, epigenetic histone modifications affect both physiological and pathological processes. In particular, cancer progression is associated with numerous epigenetic modifications. Histone demethylation can change the chromatin state, leading to the enhancement or repression of gene expression [81,82]. In the context of cancer, histone demethylation of tumor suppressor gene loci can result in gene activation and promote tumorigenesis. Thus, there has been an increased interest in developing small molecule inhibitors that target JmjC demethylases [83,84], which we will discuss below.

Recent studies have identified *N*-oxalylglycine, an analog of αKG that competitively binds at the active site of JmjC demethylases [84]. High-throughput screens indicate *N*-oxalylglycine is a KDM4E inhibitor [85]. Inhibiting KDM4E, which is often overexpressed in breast, lung, and prostate tumors, may therefore have a positive impact in cancer repression. Although *N*-oxalylglycine inhibits KDM4E, it is not an ideal compound because of its high polarity and its ability to bind to other iron (II) and αKG-dependent enzymes [86]. Therefore, it is likely to have toxicity to normal cells.

In search for an alternative to *N*-oxalyglycine, a potent inhibitor, *N*-oxalyl-d-tyrosine was found to inhibit KDM4 and KDM5 subfamilies. *N*-oxalyl-d-tyrosine is a derivative of d-tyrosine and binds to the active site of KDM4 and KDM5 subfamilies, which does not allow for αKG to bind and activate the enzymes [87]. KDM4 and KDM5 are overexpressed in various cancers and downregulate tumor suppressors. This subfamily of JmjC demethylases are amplified in lymphomas and regulate the function of the MYC oncogene in neuroblastomas [88,89]. As previously mentioned, members of the KDM5 subfamily are overexpressed in prostate cancer, thereby promoting tumorigenesis. Overall, the KDM4 and KDM5 subfamilies can be considered proto-oncogenes. Therefore, targeting these JmjC demethylases could potentially result in tumor suppression. In addition to *N*-oxalyl-d-tyrosine, pyridine dicarboxylic acids were found to completely bind to the same active site as *N*-oxalyl-d-tyrosine in KDM4 subfamilies [90]. The use of pyridine dicarboxylic acids as JmjC demethylase inhibitors has also been shown to reduce proliferation in different cancer cell lines [91]. NCDM-32B, a KDM4 small molecule inhibitor, has also been shown to reduce cell viability and anchorage-independent growth in breast cancer cell lines through regulation of multiple cancer pathways [92]. Finally, PKF118-310 is a specific KDM4A inhibitor [93]. Although the interaction between KDM4A and PKF118-310 induces apoptosis of multiple tumor types, the exact binding site is unknown. Further work on these compounds will be necessary to determine the on-target toxicity and specificity for certain JmjC demethylases.

More recently, 5-carboxy-8-hydroxyquinoline (IOX1) has been shown to inhibit a broad spectrum of JmjC demethylases by binding at the αKG binding pocket [94]. Although IOX1 directly binds the αKG binding pocket to affect JmjC demethylase activity, it also chelates iron (II), which may produce negative effects on iron-dependent proteins [94]. Additionally, the pyridine hydrazine derived from aryl *N*-heteroaryl ketones, JIB-04, has also been shown to inhibit a broad spectrum of JmjC demethylases [95,96]. Unlike IOX1, JIB-04 does not completely bind to the αKG binding pocket, but instead interacts with iron at the catalytic site [96]. Although it does not bind at the αKG binding pocket, JIB-04 still chelates iron (II) when the iron concentration is higher than 400 nM. Compared to other JmjC demethylase inhibitors that bind at the αKG binding pocket, JIB-04 tends to be more specific for JmjC histone demethylase family members as it does not interact with prolyl hydroxylases (PHDs), which require αKG for their activity [96,97]

Although numerous histone demethylase inhibitors have been characterized, they are not specific to one JmjC demethylase. Current inhibitors target JmjC demethylases by affecting the ability of αKG or iron (II) binding. The search for a histone demethylase inhibitor with higher potency and selectivity is still undergoing extensive research. Moreover, as many JmjC demethylases have multiple histone, and potentially non-histone targets, it will be important to further investigate the on-target toxicity of these inhibitors.

## 5. Conclusions

In summary, JmjC demethylases have been shown to play a role in various cancers, having both tumorigenic and tumor suppressive roles. In particular, studies have demonstrated that JmjC demethylases affect senescence and may promote tumorigenesis through suppressing the senescence-associated cell cycle arrest or activating the SASP. Interestingly, although known as histone demethylases, studies have shown that these enzymes may affect senescence through other non-histone-mediated pathways. Nevertheless, the upregulation of these enzymes in many cancers makes them a promising therapeutic target. Future work to develop more selective inhibitors and to determine on-target side effects will be necessary to delineate the therapeutic potential of inhibiting these enzymes in cancer.

## Figures and Tables

**Table 1:** Jumonji C (JmjC) histone demethylases play a role in inducing and suppressing cellular senescence.

JmjC Histone Demethylase	Target(s)	Implications in Senescence	Role in Suppressing Senescence to Promote Tumorigenesis
KDM6B	H3K27me3/2 H3K9me2/1 H4K20me1	Overexpression increases SASP gene expression [27]; promotes SAHF formation through pRb [28]; regulation of p53 [29]	Overexpressed in glioma cells to promote SASP expression [27]
KDM5B	H3K4me3/2	Silences E2F target genes [30,31]; knockdown increases H3K4 methylation at the *CDKN2A* locus [30]	
KDM5A	H3K4me3/2	Knockdown or depletion induces senescence by increasing p21, p27, p16 [32,33]	Expression is required in pRb-defective cancer [34]
KDM4A	H3K36me3/2 H3K9me3/2	Downregulation activates the p53 pathway and induces PML body accumulation [35]	Cooperates with RAS to promote transformation [35]; affects the SASP [36]
KDM4B	H3K36me3/2 H3K9me3/2	No direct evidence; p53-responsive [37,38]; important for DSB repair [38,39]	
KDM2B	H3K36me2/1 H3K4me3 H3K79me3/2	Demethylates *CDKN2A* [40] and *CDKN2B* loci [41]	Cooperates with KRAS to promote pancreatic cancer [42]; immortalizes MEFs [24,40]

SAHF: senescence-associated heterochromatin foci; SASP: senescence-associated secretory phenotype; pRB: retinoblastoma protein; DSB: double-stranded break.
